# Assessment of emergency contraceptives utilization and associated factors among female college students at Debre Tabor town

**DOI:** 10.1186/s40834-020-00139-0

**Published:** 2020-11-20

**Authors:** Tadesse Wuletaw Demissie, Araya Mesfin Nigatu, Getnet Mihretie Beyene

**Affiliations:** 1 Department of Nursing, Debre Tabor Health Science College, Debra Tabor, Ethiopia; 2 Department of Health Informatics, Institute of Public Health, University of Gondar, Gondar, Ethiopia; 3 Department of Nursing, Debre Tabor University, Debra Tabor, Ethiopia

**Keywords:** EC utilization, Ethiopia

## Abstract

**Background:**

Unwanted pregnancy is a significant public health problem worldwide. In higher education, students are exposed to the risk of unintended pregnancy, abortion, and its related negative consequences.

**Objective:**

The objective of this study was to assess the magnitude of emergency contraceptives and factors associated with its utilization among college female students at Debre Tabor Town, Northwest Ethiopia.

**Methods:**

A cross-sectional, institutional-based study was conducted from June to October 2017. A multi-stage stratified sampling technique was applied to select the study participants. Data were cleaned, coded, and entered into Epi info 7 and exported to SPSS version 20 for analysis. Bivariable and multivariable logistic regression was used to identify the association between the use of emergency contraception and the predictor variables. The *P*-value less than 0.05 at 95% CI was taken as statistical significance.

**Results:**

A total of 821 respondents participated with a response rate of 97.6%. The finding showed that 33.3% of them have used emergency contraceptives following unprotected sex. Female students’ knowledge about emergency contraceptive [AOR: 2.3; 95% CI 1.20, 4.25], age with 20–24 years category [AOR: 2.3; 95% CI 1.21, 4.49] and married [AOR: 2.8; 95% CI 1.22, 6.21] and divorced [AOR: 4.9; 95% CI 1.12, 21.08] students were found to be significant predictors of EC utilization.

**Conclusions:**

This study revealed that the level of emergency contraceptive utilization was low. Students’ level of knowledge about an emergency contraceptive, age at present, and marital status were found to be the major predictor for emergency contraceptive utilization. Therefore, responsible bodies should develop strategies that enhance the knowledge level of students at the college level on the effective utilization of emergency contraceptive methods.

## Plain English summary

Unwanted pregnancy is a significant public health problem worldwide, particularly in developing countries. In higher education, students are exposed to the risk of unintended pregnancy, abortion, and its related negative consequences.

Students were asked to use an interview-based questionnaire for their exposure and utilization of emergency contraceptives and their associated factors.

A total of 821 respondents participated with a response rate of 97.6%. The finding showed that 33.3% of them have used emergency contraceptives following unprotected sex. Female students’ knowledge about an emergency contraceptive, age with 20–24 years category, and married and divorced students were found to be significant predictors of EC utilization.

## Article summary

### Strength

College students were asked using an interview-based questionnaire for their exposure and utilization of emergency contraceptives and their associated factors.

Responsible bodies particularly college higher officials’, policymakers, and health professionals were informed to develop strategies that enhance the knowledge level of students on the effective utilization of emergency contraceptive methods; this could enhance female student’s knowledge and use of emergency contraceptives.

### Limitation

As the study was a cross-sectional egg -chicken dilemma may happen and as the reproductive issues are sensitive, students may feel shame to explain their emergency contraceptive utilization and other reproductive issues fully.

## Background

Emergency contraception also called: “post-coital contraception”, or “second chance” is a type of modern and traditional contraception that is used after unprotected sexual intercourse, following sexual abuse, misuse of regular contraception, or non-use of contraception [1].

Every year, more than 120 million couples have an unmet need for contraception and 80 million women have unintended pregnancies from which 45 million of them end up with abortion; this mainly results from unsafe sex which is the second most important risk factor for disability and death in the world’s poorest communities [2].

As it is difficult to determine the infertile time of the cycle with certainty; emergency contraceptive methods should be provided for any woman concerned about her risk of pregnancy regardless of the cycle and day of exposure; there are two types of emergency contraceptive methods: the emergency contraceptive pill (the “morning after” pill) and the intrauterine device (IUD) [3]. Hormonal emergency contraceptive pills are taken within 72 h of unprotected sexual intercourse and then 12 h late. Whereas progesterone-only pills one pill should be taken as the first dose as soon as convenient, but not later than 3 days (72 h) after unprotected intercourse to be followed by another one pill 12 h later;Levonelle has to be taken within 72 h(3 days) of un protected sex to prevent pregnancy and ellaOne(containing ulipristal acetate) has to be taken within 120 h(5 days) of sex to prevent pregnancy and the copper bearing IUCD be inserted within 5 days of un protected sex when used as an emergency contraceptive method; it is more effective than the contraceptive pill in preventing pregnancy; less than 1% of women get pregnant. The sooner the methods are used, the more effective after the act of an intended intercourse [4].

Even though, users report some side effects of emergency contraceptives like irregular bleeding, headache, nausea, weight, and mood changes [1]. If used correctly, all types of EC pills can decrease the risk of unintended pregnancy by 75% which in turn helps to reduce unplanned pregnancy and unsafe abortion [5].

A systematic review of causes of maternal mortality estimated that abortion accounted for 49% of deaths and millions more have complications; half of the deaths occur in Africa where one in four unsafe abortions is done with teenagers [6].

Despite the technological advancements in modern contraceptive methods, unintended pregnancy is still a big health problem but can be minimized by the proper utilization of emergency contraceptives. However different findings in middle and low-income countries revealed that EC utilization proportion was 29% in China, 21.2% in South Africa, 13.3% in Nigeria, 39.9% in Ghana, and 2.7% in Ethiopia [7–11].

Emergency contraceptives utilization can play an important role in reducing un planned pregnancies and thereby reducing the risk of un planned pregnancy, its associated health risks, social problem and furthermore prevents economic problems, but there were limited studies in the study area. Therefore, the objective of this study was to assess the magnitude of emergency contraceptive utilization and its associated factors among female college students at Debre Tabor Town.

## Methods

### Study design, setting, and participants

An institutional-based cross-sectional study was conducted to assess the prevalence of emergency contraceptive utilization and associated factors among female college students learning at Debre Tabor Town, Amhara Regional State, and Northwest Ethiopia. The study was conducted among three governmental and two private colleges (namely; Debre Tabor Health Science College, Debre Tabor Poly Technique College, Begemdir Teachers College, Fekede Egzi College, and Guna Tabor Business and Health Science College) found in Debre Tabor Town from June to October 2017. There were a total of 800 female students attending their education. The town is located 667 km from Addis Ababa (the capital city of Ethiopia) in the Northwest direction of the country and 97 km away from Bahir Dar city (the capital city of the region) toward the east direction.

It has also 4 kebeles (the smallest local administrative units), three public health centers, one general hospital, three medium private clinics, one primary clinic, three drug stores, and two pharmacies with an area of 3187.07 ha. It has 81, 644 estimated total population, and from which 40,985 were women. Of the 40,985 females, 19,252 were within the reproductive age at the time of the study. The estimated annual delivery number within this period was 2751 births [12].

### Sample size and sampling techniques

The sample size was determined using a single population proportion formula with the assumption of a 95% confidence interval and a margin of error of 5%. From those who had sexual experience, the proportion of students who practiced emergency contraceptive methods was assumed to be 46.3%(p) taken from the study conducted in southwest Ethiopia Mizan Tapi university brought larger sample size after considering others to have the required sample size [13]. Then, by adding a non-response rate of 10% and multiplying by a design effect of 2, a sample of 840 was determined.

A two-stage sampling technique was used; in the first case departments were selected using the lottery method and then, the sample size was allocated to each department proportional to the number of female students in each department. Finally, participant students were selected from each department proportional to their year of study using a simple random sampling technique.

### Data collection procedures

Data were collected using a self-administered Amharic version and a pretested questionnaire facilitated by two diploma nurses and one BSc nurse supervisor. It includes variables like socio-demographic characteristics, sexual and reproductive health history, knowledge, attitude, practice about EC and determinants of emergency contraceptive methods were asked.

### Data processing and analysis

Data were entered, edited, and cleaned using Epi-info version 7 and exported to SPSS version 20 for further statistical analysis. The descriptive analysis such as proportions, percentages, frequency distribution, and measure of central tendency was carried out.

Next to this, the bivariate analysis was done to identify whether there was an association between the dependent and independent variables to select the candidate variable for the multivariable analysis. Accordingly, variables found to have an association with the dependent variable less than 0.2 *p*-values were entered into multivariable binary logistic regression using the enter method for controlling the possible effects of confounders. Finally, the variables which had a significant association with p-value < 0.05 were identified as significant variables based on the odds ratio (OR), with 95% CI. The goodness of fit test was also checked.

### Operational definitions of terms

#### Emergency contraceptive utilization

Refers to methods that a woman can use to prevent pregnancy after unprotected sexual intercourse, method failure, or incorrect use.

### Data quality control measures

The quality of data was assured by giving pre-test, training for data collectors, and supervisor on the objective of the study and making frequent supervision. The completeness of the questionnaire was checked every other day by the supervisors and principal investigators.

### Ethical considerations

Ethical clearance was obtained from the Amhara Public Health Institution. Informed consent was also obtained from each study participant. Individual participant records were coded on each respective questionnaire and accessed only by the research team members to keep confidentiality.

## Results

### Socio-demographic characteristics of students

In this study, a total of 821 female college students participated in the study yielding a response rate of 97.6%. The mean age of the respondents was 19.9 (SD ±2.28) years. The youngest being 15 and the oldest 35 years old. More than one third (47.5%) of the respondents were between the age of 15–19 years. Above three fourth of (97%), the respondents were Orthodox Christianity by religion. Concerning marital status, 678 (83.7%) of the respondents were single (Table 1).

**Table 1:** Socio-demographic characteristics of female college students Debre Tabor Town northwest Ethiopia, October 2017 (*n* = 821)

Variables	Frequency	Percent
**Age (in years)**		
15–19	3390	47.5
20–24	386	47.0
> =25	45	5.5
Mean ± SD	19.9 ± 2.3	
**Marital status**		
Single	678	83.7
Married	98	11.9
Widowed	5	0.6
Divorced	27	3.3
Separated	4	0.5
**Religion**		
Orthodox	796	97.0
Muslim	20	2.4
Catholic	4	0.5
Protestant	1	0.1
**Year of Study**		
1st year	562	68.5
2nd year	149	18.1
3rd year and above	110	13.4
**Field of study**		
Health Science	302	36.8
Non-health Science	519	63.2
**Program**		
Regular	678	82.6
Extension	143	17.4

### Reproductive health characteristics

The mean age at first sexual intercourse and first pregnancies were 18.8 (SD ± 2.24) years and 19.9 (SD ± 2.6) years respectively. Eighty-nine (33.8%) of sexually active female students had had a pregnancy and 50(56.2%) of them were below the age of 20 years. Sixty-seven (75.3%) of pregnant respondents reported that their pregnancy was unplanned. Among those who reported unplanned pregnancy, 30(44.8%) failed to prevent pregnancy because of forgetting to take contraceptive methods namely oral contraceptives, and 9(13.4%) having infrequent sex. Similarly, those of untended pregnancies, 55(82.1%) of them end up with induced abortion mainly from governmental health facilities 33(60%). The major reasons for them to terminate their pregnancy were fear of discontinuing from school (50.9%) followed by fear of parents (36.4%) (Table 2).

**Table 2:** Pregnancy and related characteristics among sexually active female college students Debre Tabor Town northwest Ethiopia, October 2017

Variables	Frequency	Percent
**Ever had sex (n = 821)**		
yes	263	32.0
No	558	68.0
**Age at first sex (*n*** **= 263)**		
15–19	175	66.5
20–24	80	30.4
> =25	8	3.1
Mean (±SD)	18.8 ± 2.2	
**Ever been pregnant (n = 263)**		
Yes	89	33.8
No	174	66.2
**Ever heard about EC (n = 821)**		
Yes	456	55.5
No	365	44.5
What is your source of information		
From friends	228	50.0
From health institutions	47	10.3
From mass media	105	23.0
From boyfriends	15	3.3
From parents	17	3.7
From college teachers	44	9.6
**Age at first pregnancy (*n*** **= 89)**		
15–19	50	56.2
20–24	35	39.3
> =25	4	4.5
**Unintended pregnancy (n = 89)**		
Yes	67	75.3
No	22	24.7
**Induced abortion (*n*** **= 67)**		
Yes	55	82.1
No	12	17.9
**Place of abortion (*n*** **= 55)**		
Untrained abortionist	5	9.1
Private clinic	11	20.0
Government health institution	33	60.0
Self-infliction	6	10.9
**Reason for induced abortion (n = 55)**		
Fear of discontinuing school	28	50.9
Fear of parents	20	36.4
Economic problem	11	20.0
Since unintended	10	18.2
Stigma	7	12.7

Among the total participants, 456(55.5%) of them said that they heard information about emergency contraceptives. From these, 228 (50%) of them mentioned friends as their first source of information followed by mass media 105(23.04%) (Table 2).

### Knowledge of emergency contraceptive utilization

**Fig. 1:**
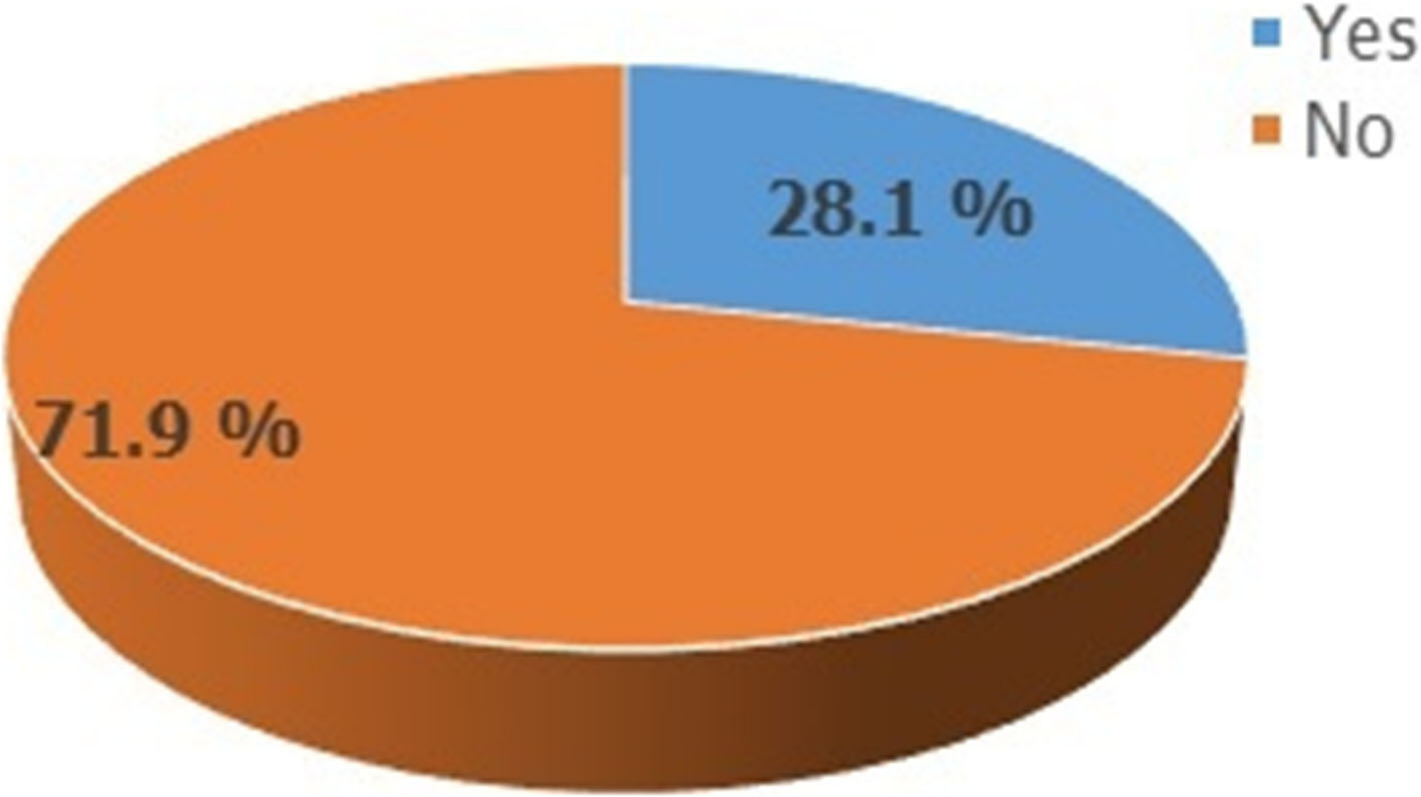
Prevalence of EC users among college female students who had a history of sexual intercourse, Debre Tabor Town, Northwest Ethiopia October 2017

Among college female students who have heard about EC 456(55.5%), only 254 (55.7%) correctly identified the time of utilization of the method, 246(53.9%) knew the recommended doses, and 169 (37.1%) recognize the recommended time interval between the doses. The index knowledge summary about EC also showed that 207(45.4%) of the respondents had good knowledge of EC (Table 3). Among 263 respondents who had a history of sexual intercourse, 74(28.1%) of them used EC as contraception (Fig. 1).

**Table 3:** knowledge about emergency contraceptive utilization among female college students in Debre Tabor Town northwest Ethiopia, October 2017 (*n* = 456)

Variables	Frequency	Percent
**Methods reported as EC**		
Oral contraceptive pills	88	19.3
IUCD	15	3.3
Injectable	25	5.5
Condoms	52	11.4
Norplant	9	2.0
Withdrawal	24	5.3
Calendar/rhythm	11	2.4
All mentioned above	147	32.2
I don’t know	88	18.6
**Mechanism of action of pills as ECs**		
Prevent ovulation	76	16.7
Prevent fertilization	73	16.0
Prevent implantation	28	6.1
Induced abortion	19	4.2
All mentioned above	82	18.0
I don’t know	178	39.0
**Can Ec cause early abortion?**		
Yes	169	37.1
No	287	62.9
**When taken early, can EC prevent STI?**		
Yes	175	38.4
No	281	61.6
**Where EC can be obtained?**		
Pharmacy	47	10.3
Governmental health institution	70	15.4
Private clinics	22	4.8
All mentioned above	237	52.0
I don’t know	80	17.5
**Recommended time to take EC pills?**		
At any time	111	24.3
Within 72 h	195	42.8
Within 5 days after sex	59	12.9
I don’t know	91	20.0
**The recommended dose of pills & IUCD**		
**respectively?**	155	34.0
One dose	91	20.0
Two and above dose	18	3.9
I don’t remember	192	42.1
I don’t know	169	37.1
**Recommended between doses of pills**		
12 h apart	27	5.9
48 h apart	89	19.5
72 h apart	171	37.5
I don’t know		
**Recommended time for IUCD as EC**		
Within 24 h after sex	54	11.8
Within 48 h after sex	11	2.4
Within 72 h after sex	39	8.6
Within 5 days after sex	65	14.3
I don’t know	287	62.9
**Effectiveness of EC pills**		
Very effective (99%)	145	31.8
Effective (75%)	41	9.0
Moderate (50%)	18	3.9
Less effective (30%)	20	4.4
I don’t know	232	50.9
**Situation(s) that EC should be taken**		
When forced to have sex	52	11.4
If condom raptured during sex	27	5.9
When there are missed pills	11	2.4
When there is sex without a contraceptive	30	6.6
All mentioned above	185	40.6
I don’t know	151	33.1
**Knowledge of EC (summary index)**		
Good knowledge	207	45.4
Poor knowledge	249	54.6

### Attitude

Three hundred sixty-four (44.3%) of the respondents had a good attitude towards EC. Four hundred ninety-one (59.8%) of the respondents replayed that EC will not cause loss of confidence between regular partners; so that 276(33.6%) of the respondents strongly agreed that EC is useful for all at risk females. On the other hand, 230 (28%) of the respondents agreed that will increase the risk of STI including HIV/AIDS (Table 4).

**Table 4:** Attitude on emergency contraceptive utilization among female college students in Debre Tabor Town northwest Ethiopia, October 2017 (n = 821)

Variables	Response			
	Strongly disagree (%)	Disagree (%)	Agree (%)	Strongly agree (%)
EC causes loose of confidence between regular partners	189(20.6)	491(59.8)	148(18.0)	13(1.6)
EC is good for all females who are at risk	210(25.6)	142(17.3)	193(23.5	276(33.6)
EC increases the risk of STI including HIV/AIDS	183(22.3)	191(23.3)	230(28.0)	217(26.4)
EC is good after unsafe sexual intercourse	192(23.4)	152(18.5)	234(28.5)	243(29.6)
EC is sign full contraceptive method	172(21.0)	272(33.1)	212(25.8)	165(20.1)
EC may cause infertility	47(5.7)	188(22.9)	250(30.5)	336(40.9)
If I had unintended sexual intercourse I would use EC	41(5.0)	80(9.7)	237(28.9)	463(56.4)
I would advise using EC for close friends if they had unintended sexual intercourse	70(8.5)	108(13.2)	313(38.1)	330(40.2)
EC promotes promiscuity	148(18)	298(36.3)	196(23.9)	179(21.8)
EC is one way of abortion	143(17.4)	173(21.1)	313(38.1)	192(23.4)
EC affect the regular contraceptive method	101(12.3)	279(34.0)	2264(32.2)	17(21.6)
Unplanned sexual intercourse is a problem for all young females	229(27.9)	390(47.2)	99(12.1)	103(12.5)
EC can be used for a long time	145(17.7)	198(24.1)	300(36.5)	178(21.7)
**Attitude (summary index)**				
Good Attitude	364 (44.3%)			
Poor Attitude	457 (55.7%)			

### Practice

**Fig. 2:**
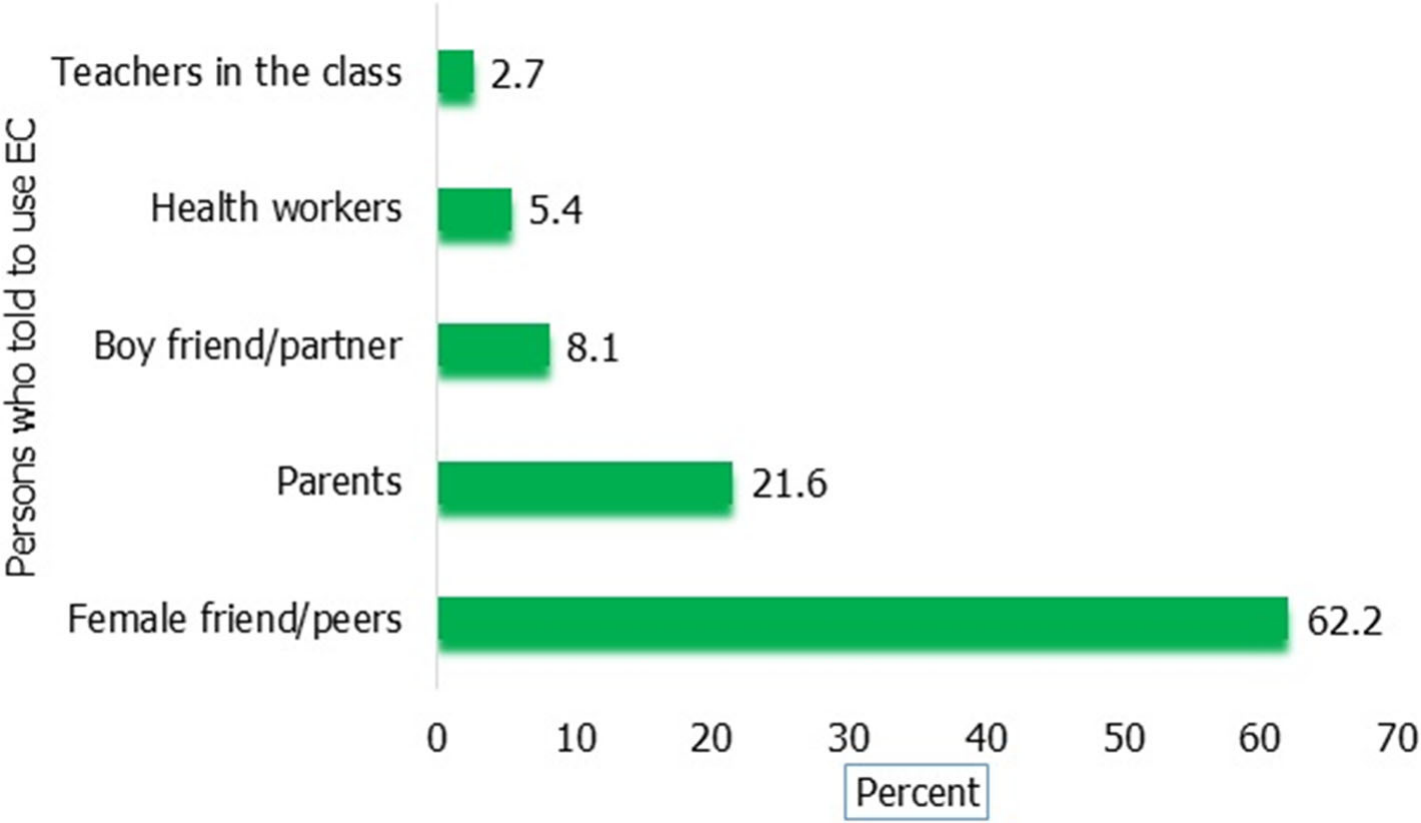
Number of students who got advice to use EC after unprotected sex, Debre Tabor Town, Northwest Ethiopia June 2017 (n = 37)

Among students who had ever been sexually active 28.1% of them used EC and from respondents who had unprotected sexual acts, 33.3% of them used EC. Oral contraceptive pills were the most common method used followed by condoms accounting for 70.3 and 18.9% respectively. However, only 48.6 and 16.2% of them correctly identified the recommended time limit (that means within 72 h. for pills and within 120 h for IUCD) respectively (Table 5). Twenty-three (62.2%) of the respondents were advised to use emergency contraceptive methods after unprotected sex by their female friends or peers (Fig. 2).

**Table 5:** Emergency contraceptive practice among female college students, Debre Tabor Town, northwest Ethiopia, October 2017

Variables	Frequency	Percent
**Ever used EC among those who had sex (n = 263)**		
yes	74	28.1
No	189	71.9
**Ever used EC among unprotected sex (*n*** **= 111)**		
Yes	37	33.3
No	74	66.7
**Methods used as EC (*n*** **= 37)**		
OC pills	26	70.3
IUCD	1	2.7
Condom	7	18.9
Withdrawal	2	5.4
Calendar	1	2.7
**Place obtained (n = 37)**		
Pharmacy	11	29.7
Government health institutions	3	8.1
Private clinics	23	62.2
**How many times used (n = 37)**		
One dose	6	16.2
Two and above	18	48.6
I don’t remember	1	2.7
As necessary	12	32.4
**Time EC was used (n = 55)**		
Within 72 h for pills	14	37.8
Within 120 h for IUCD	12	32.4
I used as I want	10	27.0
I don’t remember	1	2.7

### Determinants of emergency contraceptive utilization

The multivariable logistics regression analysis showed that college female students whose age category between 20 and 24 years (AOR = 2.3, 95% CI: 1.21, 4.49) were two times more likely to use EC as a contraception method. Married students (AOR = 2.8, 95% CI: 1.22, 6.21) and divorced once (AOR = 4.9, 95% CI: 1.12, 21.08) were two and five times more likely to use EC as compared to unmarried students respectively. On the other hand, students who had good knowledge (AOR = 2.3, 95% CI: 1.20, 4.25) were two times more likely to use EC as a contraception option (Table 6).

**Table 6:** Bivariate and multivariate analysis on emergency contraceptive utilization among female college students in Debre Tabor Town northwest Ethiopia, October 2017 (n = 263)

Variables	EC used		Crude OR (95% CI)	Adjusted OR (95% CI)
	No (%)	Yes (%)		
Age (in years)				
15–19	91(48.2)	21(28.41)	1.00	1.00
20–24	90(47.6)	45(60.8)	2.2(1.19, 3.93)	2.3(1.21, 4.49)*
> =25	8(4.2)	8(10.8)	4.3(1.46, 12.87)	1.9(0.52, 7.32)
Marital Status				
Never married	169(89.4)	49(66.2)	1.00	**1.00**
Married	16(8.5)	19(25.3)	4.1(1.96, 8.56)	2.8(1.22, 6.21)*
Divorced	4(2.1)	6(8.1)	5.2(1.40, 19.07)	4.9(1.12, 21.08)*
Extra Job				
Yes	11(5.8)	12(16.2)	3.1(1.32, 3.46)	2.5(0.98, 6.62)
No	178(94.2)	62(83.8)	1.00	1.00
Ever Pregnant				
Yes	6(3.2)	14(18.9)	7.1(2.62, 19.34)	3.7(0.50, 27.99)
No	183(96.8)	60(81.1)	1.00	
Unintended pregnancy				
Yes	4(2.1)	11 (14.9)	8.1(2.48, 26.27)	1.7(0.16, 17.17)
No	185(97.9)	63(81.1)	1.00	01.0
Field of Study				
Non-Health science	91(48.1)	25(33.8)	1.00	1.00
Health science	98(51.9)	49(66.2)	1.8(1.04, 3.19)	1.3(0.69, 2.49)
Knowledge				
Good knowledge	79(41.8)	47(63.5)	2.4(1.39, 4.22)	2.3(1.20, 4.25)*
Poor knowledge	110(58.2)	27(36.5)	1.00	

N.B: 1 = reference,*= significantely associated factor Hosmier and lemesho Goodness of fit test = 0.591

## Discussion

This study result showed that the utilization of EC was low. Knowledge of female students, age at present, and the marital status of the students were the major predictors of EC utilization.

In this study, we found that 33.3% (95% CI: 25.2, 42.3%) of those who had unprotected sexual act used EC, which is similar to the report of the studies done at Adama University (Ethiopia) 26.7% [14], Ghana 39.9% [10] and China 29% [11], but was lower than studies done in Ethiopian universities like Mizan Tepi 46.3% [13] and Adds Ababa 75% [15]. The difference might be due to low awareness, accessibility of EC methods, being in campus dormitory for those university students whereas in the private dormitory for college students; there is gender base and sexual health education in the colleges and universities by using gender clubs and clinics in the universities,but there may be difference in performance among the organizations. However, our study was higher than the study done in Ethiopia like Adama University 4.7% [16], Hawasa 10.8% [17], Jima 6.8% [18] and abroad Nigeria (10 &13.3%) [8, 19] and South Africa 21.2% [7]. The possible explanation for this might be a difference in socio-demographic and cultural background characteristics of the respondents and time-lapse.

A statistically significant association was obtained between the age of respondents and their EC utilization. Respondents whose age category is 20–24 years (AOR = 2.3, 95% CI: 1.21, 4.49) were two times more likely to use EC as contraception compared to those within the age range of 15–19 years old. The finding is similar to studies done in Ethiopia [13, 14, 16] and South Africa [7]. The probable reason for this could be younger students might have less information about the proper use of EC due to the fact that they were newly enrolled in the college and might not have received information in their prior schooling.

Another statistically significant association was also obtained between marital status and emergency contraceptive utilization of students. Respondents who were ever married (AOR = 2.8, 95% CI: 1.22, 1.21) and divorced (AOR = 4.9, 95% CI: 1.12, 21.08) were three and five times more likely to use emergency contraceptive respectively as compared to single ones. The finding is similar to studies done at Adama University (Ethiopia) [14, 16]. The possible justification for this might be access to current information; married students might get current information on EC from their partners. Besides, the effect of marital status and increment in age on EC utilization could also be linked to issues like minimizing the fear of being seen by others.

Finally, another significant association was also found between respondents having good knowledge of EC and EC utilization. Respondents who had good knowledge (AOR = 2.3, 95% CI: 1.20, 4.25) were two times more likely to use emergency contraceptives as compared to those having poor knowledge. This finding was consistent with studies conducted in Ethiopian Universities like Mizan Tepi [13], Arba Minch [20], and Adama [14]. The possible reason for this might be a good knowledge. Students who had good knowledge would help them to identify where, when, and how to use EC in preventing unintended pregnancy and abortion that could result from unprotected sex.

Although the finding of this study may not be generalized to students who are out of colleges, it has demonstrated the sexual and reproductive health problems faced by students in colleges found at Debre Tabor. Since an anonymous self-administered questionnaire was used, the possibility of social desirability bias cannot be eliminated as the study touches sensitive issues.

## Conclusion

The study finding pointed out that the proportion of emergency contraceptive utilization among female college students was low. Knowledge of students on EC, age at present, and marital status were found to be major determinants. Educating adolescents about emergency contraceptives to give attention to available methods, correct time of use, and promoting emergency contraceptives using formal and informal education like health education sessions gender club sensitization and peer education about sexual health and contraception so as to increase knowledge on EC and to enhance utilization and to make emergency contraceptive methods readily accessible.

## Data Availability

The original data are available at hand and maybe delivered upon request via the corresponding author.

## Abbreviations

EC: Emergency Contraceptive
HIV/AIDS: Human Immune Deficiency Virus/Acquired Immune Deficiency Syndrome
IUCD: Intra-Uterine Contraceptive device N.B: 1.00=reference, * =significantly associated factor Hosmier and Lemesho Goodness of fit test=0.591
STI: Sexually Transmitted Infection
